# Reversible Pulmonary Arterial Hypertension Due to Fenfluramine in a Young Child: A Case Report

**DOI:** 10.7759/cureus.108354

**Published:** 2026-05-06

**Authors:** Uzoma Ndukwe, Adrianne Parkey, Debopam Samanta, Abdelrahman Masri, Erin Willis

**Affiliations:** 1 Pediatrics, University of Arkansas for Medical Sciences, Little Rock, USA; 2 Pediatric Neurology, University of Arkansas for Medical Sciences, Little Rock, USA; 3 Pediatric Cardiology, University of Arkansas for Medical Sciences, Little Rock, USA

**Keywords:** echocardiogram, fenfluramine, pulmonary arterial disease, seizures, young child

## Abstract

Fenfluramine is approved for Dravet syndrome and Lennox-Gastaut syndrome (LGS) in children under two years of age and is increasingly used off-label for developmental and epileptic encephalopathies (DEEs). Due to the risk of pulmonary arterial hypertension (PAH) and valvular disease, serial echocardiographic monitoring is required. While fenfluramine-associated cardiac toxicity is well described in adults, data in pediatric patients, especially those under two years of age, remain limited.

We report a child under two years of age with SCN1B-related DEE who developed asymptomatic fenfluramine-associated PAH after one year of treatment. Fenfluramine resulted in marked seizure reduction but was discontinued after the detection of PAH. Subsequent echocardiography demonstrated resolution of PAH, accompanied by worsening seizure burden.

This case emphasizes the rare occurrence of fenfluramine-associated PAH in children under two years of age and underscores the importance of vigilant cardiac surveillance in this population.

## Introduction

Pathogenic SCN1B variants, which encode the β1 subunit of voltage-gated sodium channels involved in neuronal excitability and network development, have been described in association with early-onset, drug-resistant epilepsy accompanied by developmental delay or regression [1,2]. Despite increasing availability of anti-seizure medications, most patients with Lennox-Gastaut syndrome (LGS), Dravet syndrome, and other developmental and epileptic encephalopathies (DEEs) continue to have intractable seizures, with an associated increased risk of sudden unexpected death in epilepsy (SUDEP) and decreased quality of life [1,3].

For this reason, medications with novel mechanisms of action, such as fenfluramine, are being increasingly used off-label despite known cardiac risks. A recent comprehensive scoping review of fenfluramine confirmed robust reductions in generalized tonic-clonic seizures across various DEEs, including SCN1B-related disorders [4]. Fenfluramine’s anticonvulsant activity is mediated by modulation of serotonergic and sigma-1 receptors [5].

Serotonin levels have been reported to be elevated in pulmonary arterial hypertension (PAH), and serotonin acts as a growth factor on pulmonary artery smooth muscle cells, possibly leading to the development of PAH [6]. Historically, fenfluramine has been causally linked to PAH and valvular heart disease (VHD) in adults [5,6].

In pediatric populations aged two years or older, multiple randomized controlled trials (RCTs) and long-term open-label extension (OLE) studies in Dravet syndrome and LGS report a low incidence of PAH or VHD with standardized echocardiographic monitoring [7,8]. Nonetheless, rare case reports of pediatric fenfluramine-associated PAH exist, including a four-year-old with LGS who developed asymptomatic PAH, which resolved after fenfluramine discontinuation and did not recur with later reintroduction [9].

Although fenfluramine is increasingly used in the treatment of drug-resistant epilepsy, its cardiac risk profile in very young children under two years of age is not well characterized. To our knowledge, this case report is the first to document the development of reversible fenfluramine-associated PAH in a patient under two years of age.

## Case presentation

**Figure 1:**
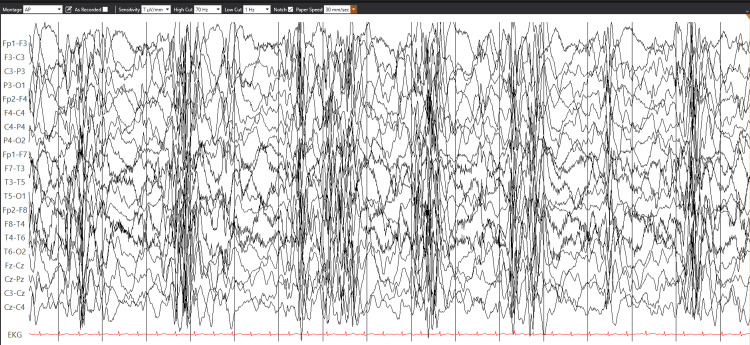
EEG with a background consistent with hypsarrhythmia/epileptic encephalopathy EEG: Electroencephalogram

**Figure 2:**
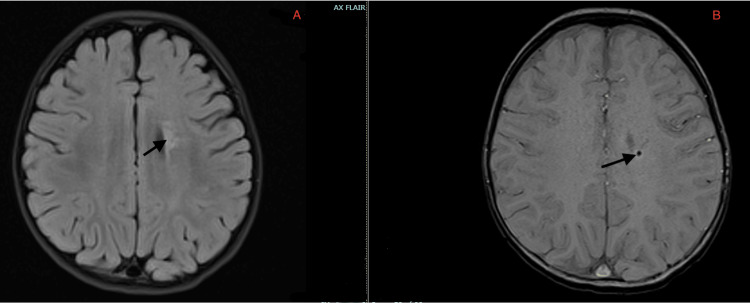
MRI brain (A) Arrow shows a very small left periventricular FLAIR change (bright spot); (B) Arrow shows a small amount of hemosiderin deposition. FLAIR: Fluid-Attenuated Inversion Recovery; MRI: Magnetic Resonance Imaging

A 17-month-old female with no significant past medical history and an uneventful birth history presented with multiple seizure types, including epileptic spasms, tonic, and atonic seizures. Parents reported that she had met all her milestones; however, she had shown some regression in the days prior to presentation. Video electroencephalogram (EEG) recording showed a background consistent with hypsarrhythmia (Figure 1), with a BASED score of 5 [10]. MRI of the brain showed a small area of gliosis and hemosiderin staining in the left periventricular white matter (Figure 2).

**Figure 3:**
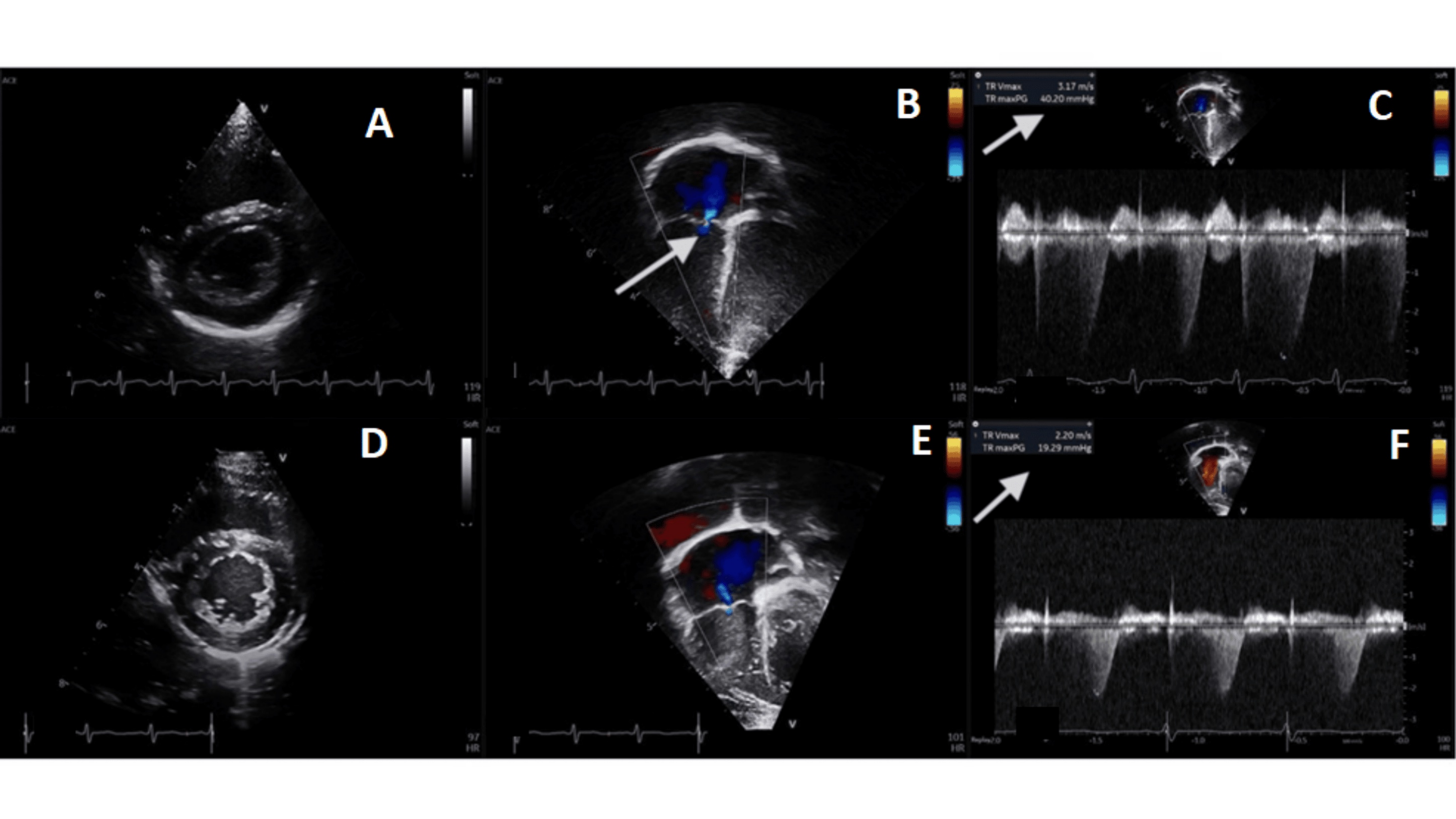
Echocardiographic comparison at different points of clinical course (A-C) Some loss of interventricular septum symmetry in A compared to D, normal right ventricular cavity, and trivial tricuspid valve insufficiency (arrow in B), with elevated tricuspid regurgitant velocity and tricuspid regurgitant pressure gradient (arrow in C). Features suggestive of PAH. (D-F) Normal right ventricular cavity, normal tricuspid valve, normal tricuspid regurgitant velocity, and normal tricuspid regurgitant pressure gradient (arrow in F). PAH: Pulmonary Arterial Hypertension

An EpiXpanded panel genetic testing was obtained, which revealed a mutation in the SCN1B gene associated with epileptic encephalopathy, and she was diagnosed with SCN1B-related DEE. She was initially treated with high-dose steroids and levetiracetam, which improved but did not resolve her spasms. Clobazam was subsequently added due to worsening tonic seizures. Repeat video EEG showed some improvement of the background to a BASED score of 2 [10]; however, she continued to have frequent spasms, tonic seizures, and atonic seizures occurring multiple times per day. At 20 months of age, she was started on fenfluramine and titrated to a target dose of 0.7 mg/kg/day, with a marked reduction in seizure frequency of >50%. Her other anti-seizure medications were continued while the steroid was tapered. The baseline echocardiogram (ECHO) prior to starting treatment, per protocol, was normal, and the follow-up ECHO after four months of therapy was also normal. ECHO at 11 months of therapy showed new PAH with an elevated right ventricular systolic pressure estimate of 45.2 mmHg but no valvular disease (Figures 3A-3C). Her mother was notified, and the decision was made to wean her off the medication over one week. Follow-up ECHO five months after discontinuing fenfluramine showed resolution of PAH, with right ventricular systolic pressure estimated at 25.4 mmHg (Figures 3D-3F). Unfortunately, seizures worsened to 10-20 times per day, despite the addition of both cannabidiol and divalproex sodium. Due to robust seizure response and resolution of PAH, the family is considering reintroducing fenfluramine at a lower dose, but there is little data to guide weighing the risks and benefits of this decision. 

## Discussion

DEEs like SCN1B-related DEE are characterized by multiple seizure types, developmental delays or regression, and failure of multiple anti-seizure medications [1,11]. Due to increased risk of SUDEP, patients will often try multiple anti-seizure medications with the goal of improving seizure burden and quality of life, despite known risks. A comprehensive case series of SCN1B DEE reported an excellent clinical response to fenfluramine, resulting in a 50% reduction in seizures, status epilepticus, and generalized tonic-clonic seizures [2]. For this reason, her family consented to the off-label use of fenfluramine, with excellent clinical response. While many typical seizure medications affect the GABA or glutamate balance, fenfluramine’s mechanism involves serotonergic modulation of the 5-HT and sigma-1 receptors [6,11]. In DS and LGS trials, fenfluramine adjunctive therapy showed clinically meaningful reductions in convulsive or drop seizures [3,7,8,12]. A 2024 scoping review across DEEs, including SCN1B-related disorders, observed median reductions in generalized tonic-clonic or tonic-clonic seizures ranging from ~47% to 100% in selected cohorts, and ~72% of patients in 10 studies achieved ≥50% reduction [4]. Additionally, in DS, a time-to-event analysis showed that fenfluramine increases the interval until the next seizure, resulting in more seizure-free days, which translates to improved quality of life [3]. 

With fenfluramine use (0.2-0.7 mg/kg/day), published RCTs and OLEs have not reported clinically significant VHD or PAH in patients aged two years or older [7,8,12]. For example, in the Dravet OLE involving 327 patients over a median of 23.9 months, no cases of PAH or VHD were observed via serial echocardiography [8]. Similarly, interim OLE data in LGS up to ~12 months (median exposure, 364 days) found no echocardiographic evidence of valvulopathy or PAH [12].

However, absence of evidence does not mean absence of risk. Rare occurrences of pediatric fenfluramine-associated PAH exist. The only documented case report in the literature is a four-year-old with LGS who developed asymptomatic PAH and tricuspid regurgitation after six months of therapy, and subsequent cessation led to reversal of PAH, after which the patient was able to tolerate fenfluramine again without PAH recurrence [9]. Our case adds to the literature by demonstrating fenfluramine-associated PAH in a very young patient under two years of age, who also had resolution after withdrawal of the medication. Unfortunately, seizures worsened to 10-20 times per day, despite the addition of other medications. Due to the robust seizure response and resolution of PAH, the family is considering reintroducing fenfluramine at a lower dose, but there is little data to guide the weighing of risks and benefits of this decision.

In our patient, despite the very young age and off-label use, fenfluramine produced a meaningful seizure reduction, consistent with its known efficacy profile in older children [7,8,12]. In DEEs such as SCN1B-related disorders, there is high morbidity and mortality and limited therapeutic options; therefore, the benefits of fenfluramine may outweigh the risks, especially when patients are monitored carefully and there is evidence that the cardiac side effects are reversible. Our case highlights that although many large studies have shown an absence of cardiac risks in the pediatric population, risks still exist. However, like the only other pediatric case published [9], our patient was asymptomatic and showed reversal of PAH with discontinuation of therapy. Given that young children cannot reliably verbalize symptoms like dyspnea or fatigue, cardiac monitoring is even more critical in this age group. The fact that the PAH reversed suggests a likely functional or early vascular remodeling phenomenon rather than irreversible damage, aligning with previously reported reversibility in pediatric cases [5,9]. To mitigate risk in very young patients or those who are nonverbal, more frequent ECHO surveillance could be considered until more safety data are accrued. This might make it feasible to reintroduce fenfluramine at a lower dose.

Emerging analogs, such as bexicaserin (LP352), with a similar mechanism of action but lower cardiac risk, are promising for this vulnerable group, although the clinical data are not yet fully validated [13]. 

## Conclusions

Our index case describes a child under two years of age with SCN1B-related DEE who developed fenfluramine-associated PAH that resolved after withdrawal of the medication. Children at this age cannot verbalize the symptoms of PAH well; therefore, more frequent screening with ECHOs could be considered. If PAH develops in young children, it is likely reversible after withdrawal of fenfluramine. Careful discussion should be held with families when using fenfluramine off-label for DEE, weighing the risks and benefits. Fenfluramine should be discontinued if PAH is detected, although the timeline for doing so is not well established. If the decision is made to reintroduce fenfluramine later, it should be done cautiously at a lower dose, with close echocardiographic follow-up. More studies are needed to document the development of fenfluramine-associated PAH in children to help guide the safety of reintroducing fenfluramine once PAH has developed and subsequently resolved. 
